# Screening by Health Care Systems for Barriers to Patient Engagement With Digital Health Care: Cross-Sectional Survey Study

**DOI:** 10.2196/85205

**Published:** 2026-02-25

**Authors:** Jonathan J Shih, Andersen Yang, Vivian E Kwok, Amy R Sheon, Robert L Ellis, Emilia H De Marchis, Lisa C Diamond, Marika Dy, Courtney R Lyles, Carmen Ma, Nilpa D Shah, Kelsey H Natsuhara, Sarah B Rahman, Jorge A Rodriguez, Urmimala Sarkar, Anjana E Sharma, Elaine C Khoong

**Affiliations:** 1 School of Medicine University of California, San Francisco San Francisco, CA United States; 2 Division of General Internal Medicine at Zuckerberg San Francisco General Hospital Department of Medicine University of California, San Francisco San Francisco, CA United States; 3 Action Research Center for Health University of California, San Francisco San Francisco, CA United States; 4 Public Health Innovators, LLC North Bethesda, MD United States; 5 Center for Healthcare Policy and Research University of California, Davis Sacramento, CA United States; 6 Department of Family and Community Medicine University of California, San Francisco San Francisco, CA United States; 7 Hospital Medicine Service, Department of Medicine Memorial Sloan Kettering Cancer Center New York, NY United States; 8 Immigrant Health and Cancer Disparities Service, Department of Psychiatry and Behavioral Sciences Memorial Sloan Kettering Cancer Center New York, NY United States; 9 Department of Psychiatry & Behavioral Sciences University of California, San Francisco San Francisco, CA United States; 10 Division of Hematology and Oncology Department of Medicine University of California, San Francisco San Francisco, CA United States; 11 Alameda Health System Oakland, CA United States; 12 Division of General Internal Medicine and Primary Care Brigham and Women's Hospital Boston, MA United States; 13 Division of Clinical Informatics and Digital Transformation at Zuckerberg San Francisco General Hospital Department of Medicine University of California, San Francisco San Francisco, CA United States

**Keywords:** health equity, telemedicine, health care disparities, health information technology, social drivers of health

## Abstract

**Background:**

Digital health tools, including patient portals, telemedicine, and mobile health apps, are increasingly a core part of health care. Digital readiness, encompassing both digital access and literacy, is crucial for enabling patients to effectively engage with the increasing number of digital health tools. Despite growing recognition of digital readiness as a health-related social need, little is known about digital readiness screening practices.

**Objective:**

We aimed to assess the extent of digital readiness screening and the organizational factors associated with screening.

**Methods:**

From January to May 2024, we administered an online survey to a convenience sample of clinicians or informatics leaders from US health care systems. Our primary outcome was whether the respondent reported that their organization screened for digital readiness (yes vs no), and the secondary outcome was self-reported barriers to screening. We asked respondents to report characteristics related to their health system, including health system type, geographic area, payers accepted, patient population characteristics, screening practices for health-related social needs (eg, screening for food insecurity), and awareness of digital inclusion policies and programs. Using bivariate logistic regression models, we examined organizational characteristics associated with screening for digital readiness.

**Results:**

Of 144 total respondents, 64 (44%) reported screening patients for digital readiness. Organizations serving uninsured patients had lower odds of screening (odds ratio [OR] 0.32, 95% CI 0.14-0.72). Less than half of respondents to the digital readiness survey (47/99, 47%) were familiar with any digital readiness–related policy, but screening was more likely when respondents were familiar with at least one policy or program promoting equitable digital readiness (OR 6.6, 95% CI 2.4-20.6). Screening for other health-related social needs was not associated with digital readiness screening. The most frequently cited barriers to screening for digital readiness were lack of resources to address digital access (n=45, 45%), lack of resources to implement screening (n=42, 42%), and lack of time (n=41, 41%).

**Conclusions:**

Digital readiness screening has had limited adoption in US health care systems, particularly in settings serving the populations most likely to experience challenges with digital access or literacy. The limited adoption of digital readiness screening likely reflects lower awareness of digital readiness as a social need and a lack of infrastructure to support its uptake, such as standardized screening questions or a workforce trained on how to screen for and intervene on barriers to digital readiness. Low awareness of digital equity policies that might incentivize digital readiness screening further hinders adoption. Without increased adoption of digital readiness screening and/or interventions to mitigate barriers to digital readiness, digital health tools are unlikely to be accessible to or benefit all populations. Multilevel interventions, including policy changes and workforce training, are likely necessary to increase the adoption of digital readiness screening and mitigation efforts that address barriers to digital exclusion.

## Introduction

Digital inclusion is the availability of digital technology and the ability to use it [1]. As health care systems increasingly use patient portals, telehealth, and remote monitoring, digital inclusion has become essential for equitable access to care and patient engagement [2,3]. It has also been identified as a “super social determinant of health,” due to its influence on other key determinants, such as education and employment [4,5]. Consequently, barriers to patient engagement with digital tools, including limited device availability, internet connectivity, and digital literacy, threaten health equity [6,7].

To support digital inclusion, several policies and programs (eg, the Affordable Connectivity Program) have aimed to increase broadband infrastructure, access to devices, and systematic assessment and mitigation of digital inclusion barriers. These programs align with broader trends to intervene on health-related social needs (HRSNs) [8]. Efforts to “screen-and-intervene” on HRSNs have mostly been directed toward areas such as food and housing insecurity and have aimed to improve health outcomes by linking patients with resources [9]. However, these efforts have largely excluded screening for barriers to patient engagement with digital health tools, referred to here as “digital readiness,” which encompasses some but not all elements of digital inclusion (eg, technical support and inclusive tool design). Digital readiness screening includes identifying barriers to engagement with digital health tools, such as access (device ownership and internet access) and literacy (digital skills and confidence) [10-13], and serves as a critical first step toward equitable digital health use.

Despite the increasing importance of digital health tools, little is known about digital readiness screening in US health care systems. To address this gap, we surveyed health care institutions to assess the extent of digital readiness screening and the organizational factors associated with screening.

## Methods

### Study Design and Recruitment

We conducted a cross-sectional survey using a convenience sample of health system leaders, clinicians, staff, and informatics personnel working in ambulatory care in the United States. From January to May 2024, we disseminated a Qualtrics survey. We emailed ambulatory practice–based research networks identified on the Agency for Healthcare Research and Quality’s Practice-based Research Networks Registry website [14] to ask if they would distribute our survey. We also disseminated the survey link through social media and professional societies (such as those for medical informatics, general internists, and family physicians). We included individuals who could report their organization’s ambulatory digital readiness screening practices and excluded invalid responses (eg, duplicate or bot-generated responses). We collaborated with another study team to include our digital readiness questions in the digital navigation survey (the supplementary survey) distributed to California safety-net systems. As a result, our sample included responses collected by their study and our own. (Details of the supplementary survey were previously published [15].) Additional methodological details appear in Multimedia Appendix 1.

### Ethical Considerations

The University of California, San Francisco Institutional Review Board approved our study (23-39284) with a waiver of informed consent. We stored data on secure institutional servers and deidentified the data for reporting. Participants received a US $10 gift card upon completing the survey.

### Survey Development

Our survey (Multimedia Appendix 1) assessed digital readiness screening practices, barriers to screening, and factors associated with screening. Questions were adapted from validated surveys [16,17] combined with newly developed questions tailored to our study objectives.

### Variables

The primary outcome was whether the organization screened for digital readiness, dichotomized as “yes” vs “no” (“no” collapsed the responses “no,” “don’t know,” and “not currently but planning to”). As a secondary outcome, we assessed self-reported barriers to screening, a variable captured only in our digital readiness survey and not in the digital navigation survey.

Organizational characteristics were used as predictor variables and included health system type (academic, not-for-profit, for-profit, nonfederal governmental, federally qualified health center [FQHC], or Veterans Affairs); geographic area (rural, suburban, or urban); accepted payers (Medicaid, traditional Medicare, Medicare Advantage [MA], commercial insurance, city or county safety-net insurance, and uninsured or self-pay); and patient population characteristics, including the proportion of patients with limited English proficiency (LEP) (0% to <25%, 25% to <50%, or ≥50%) and the proportion identifying as racial and ethnic minorities (0% to <25%, 25% to <50%, or ≥50%).

In our digital readiness survey, we captured 2 additional sets of predictor variables: HRSN screening practices and awareness of digital inclusion policies and programs. We asked whether the organization screened for HRSNs (similarly to the primary outcome, responses were dichotomized as yes vs no), which of 9 HRSNs they screened for, and their awareness of the following policies and programs: the Affordable Connectivity Program (ACP) of the Federal Communications Commission (FCC) [18,19], Healthy People 2030 health literacy objectives [20], Joint Commission requirements for reducing disparities,[21] MA digital literacy screening requirements [22], Digital Equity Act [23], and Medicare Payment for Community Health Workers [24]. Policy awareness was dichotomized as familiar vs not familiar (collapsed the responses “have heard of it but not familiar” and “have not heard of it”).

### Statistical Analysis

Associations between each predictor variable (organizational characteristics, HRSN screening, and policy awareness) and the primary outcome were assessed using chi-square analyses and unadjusted bivariate logistic regression models. For each association, we calculated the odds ratio (OR) and corresponding 95% CI, with significance defined as *P*<.05. We conducted a sensitivity analysis using an alternative grouping for the outcome variable (“yes” or “not currently but planning to” vs “no” or “don’t know”).

## Results

### Organization Characteristics

Our total sample included 144 respondents (Table 1), of which 99 were from our digital readiness survey and 45 from the supplementary survey. The most common organization types were not-for-profit (31%), academic (29%), and FQHCs (28%). Most respondents served urban areas (67%) and patient populations where <25% had LEP (40%). More than one-third (35%) reported that a majority of their patients identified as racial/ethnic minorities. There was payer diversity, with strong representation from organizations that cared for Medicaid-insured (72%), uninsured or self-pay (61%), and city or county safety-net insured patients (41%). Most (60%) organizations accepted MA. Respondents represented organizations from all 50 states.

**Table 1:** Characteristics of screening among overall sample (N=144). Overall, 64 of 144 (44%) respondents currently screened for digital readiness.

Characteristic		Respondents^a^, n/N (%)	Respondents that currently screened for digital readiness, n/N (%)
**Type of health system**			
	Not-for-profit	45/144 (31)	19/45 (42)
	Academic	42/144 (29)	16/42 (38)
	Federally qualified health center	40/144 (28)	14/40 (35)
	Nonfederal governmental (eg, state or county funded)	19/144 (13)	7/19 (37)
	For-profit	18/144 (13)	11/18 (61)
	Veteran Affairs (VA)	6/144 (4)	5/6 (83)
	Other	5/144 (4)	2/5 (40)
**Location**			
	Urban	96/144 (67)	41/96 (43)
	Suburban	52/144 (36)	20/52 (39)
	Rural	33/144 (23)	13/33 (39)
**Payers accepted**			
	Medicaid	104/144 (72)	39/104 (38)
	Traditional Medicare	101/144 (70)	37/101 (37)
	Commercial insurance plan	90/144 (63)	32/90 (36)
	Uninsured/self-pay^b^	88/144 (61)	29/88 (33)
	Medicare Advantage plan	87/144 (60)	37/87 (43)
	City or county safety-net insurance	59/144 (41)	19/59 (32)
	Other	10/144 (7)	3/10 (30)
	Don’t know	3/144 (2)	0/3 (0)
**Patients with limited English language proficiency (%)**			
	0 to <25	58/144 (40)	23/58 (40)
	25 to <50	29/144 (20)	13/29 (45)
	≥50	24/144 (17)	9/24 (38)
	Unsure/don’t know	8/144 (6)	3/8 (38)
**Patients that identify as racial/ethnic minority individuals (%)**			
	0 to <25%	40/144 (28)	17/40 (43)
	25 to <50%	21/144 (15)	9/21 (43)
	≥50%	50/144 (35)	21/50 (42)
	Unsure/don’t know	8/144 (6)	1/8 (13)

^a^Percentages are calculated using the full sample of 144 respondents. Missing responses ranged from 19 to 25 across variables: health system type (n=19), location (n=21), accepted payers (n=23), and patient demographics (n=25). Categories within health system type, location, and payers accepted were not mutually exclusive, so sum of counts may exceed the total sample size.

^b^*P*<.05 for association with screening in bivariate analysis.

### Digital Readiness Screening and Organizational Predictors of Screening

Among the 144 respondents, 64 (44%) reported that their organization screened for barriers to digital readiness. Organizations serving uninsured patients had lower odds of screening compared to those that did not (OR 0.32, 95% CI 0.14-0.72). Digital readiness screening was not associated with health system type, location, or patient population characteristics (ie, LEP or racial/ethnic minority status).

### HRSN Screening, Digital Inclusion Policy Awareness, and Association With Digital Readiness Screening

Among the 99 digital readiness survey respondents asked about HRSN screening practices and policy awareness, 57 (58%) reported screening for at least one HRSN (Table 2). These organizations most frequently screened for food (51/57, 89%) and housing (48/57, 84%) insecurity. Almost half (47/99, 47%) of all organizations were familiar with any digital readiness–related policy. The greatest familiarity was with the FCC’s ACP (24%) and the least with the MA digital literacy screening requirements and the Digital Equity Act (17%). Among the 56 respondents whose organizations accepted MA, only 12 (21%) were familiar with the MA digital literacy screening requirement.

HRSN screening was not associated with digital readiness screening. Familiarity with any of the 6 policies was associated with screening (OR 6.6, 95% CI 2.4-20.6). Familiarity with the following individual policies was associated with screening: the FCC’s ACP (OR 5.6, 95% CI 2.0-16.8), Joint Commission disparities requirement (OR 3.5, 95% CI 1.2-10.5), MA screening requirement (OR 3.2, 95% CI 1.1-10.4), and the Digital Equity Act (OR 4.5, 95% CI 1.5-15.6).

Sensitivity analyses yielded similar results (Multimedia Appendix 1, Tables S1 and S2), with additional associations between utility screening and Healthy People 2030 familiarity and digital readiness screening. Caring for commercially insured patients was associated with lower odds of screening.

**Table 2:** Characteristics of health-related social need (HRSN) screening and familiarity with digital readiness policy (N=99). Overall, 47 of 99 (48%) respondents currently screened for digital readiness.

Characteristic		Respondents^a^, n/N (%)	Respondents that currently screened for digital readiness, n/N (%)
**Currently screening for HRSNs**			
	Yes	57/99 (58)	29/57 (51)
	No	17/99 (17)	6/17 (35)
**Screening for other HRSN items^b^**			
	Food insecurity or hunger	51/57 (89)	24/51 (47)
	Housing (instability, quality, or financing)	48/57 (84)	23/48 (48)
	Interpersonal violence	46/57 (81)	21/46 (46)
	Transportation	39/57 (68)	18/39 (46)
	Employment and income	35/57 (61)	17/35 (49)
	Social isolation (lack of family and social support)	31/57 (54)	16/31 (52)
	Education	29/57 (51)	13/29 (45)
	Utility needs	29/57 (51)	17/29 (59)
	Other	3/57 (5)	2/3 (67)
**Level of familiarity with any of the 6 digital inclusion policies**			
	Familiar with any policy details^c^	47/99 (47)	28/47 (60)
	Not familiar with any policy details	33/99 (33)	6/33 (18)
**Level of familiarity with the Federal Communications Commission’s Affordable Connectivity Program**			
	Familiar with the policy details^c^	24/99 (24)	17/24 (71)
	Not familiar with the policy details	56/99 (57)	17/56 (30)
**Level of familiarity with the Joint Commission requirements to reduce health care disparities**			
	Familiar with the policy details^c^	20/99 (20)	13/20 (65)
	Not familiar with the policy details	60/99 (61)	21/60 (35)
**Level of familiarity with the Medicare Physician Fee Schedule payment for community health integration services by community health workers**			
	Familiar with the policy details	20/99 (20)	10/20 (50)
	Not familiar with the policy details	60/99 (61)	24/60 (40)
**Level of familiarity with the Healthy People 2030 health literacy objectives focused on health communication and information technology**			
	Familiar with the policy details	18/99 (18)	11/18 (61)
	Not familiar with the policy details	62/99 (63)	23/62 (37)
**Level of familiarity with the Digital Equity Act**			
	Familiar with the policy details^c^	17/99 (17)	12/17 (71)
	Not familiar with the policy details	63/99 (64)	22/63 (35)
**Level of familiarity with the Medicare Advantage digital literacy screening requirements**			
	Familiar with the policy details^c^	17/99 (17)	11/17 (65)
	Not familiar with the policy details	63/99 (64)	23/63 (37)

^a^Percentages are calculated using all 99 respondents who received these questions. Missing data ranged from 19 to 25 across items: policy familiarity (n=19) and HRSN screening behaviors (n=25). HRSN screening categories were not mutually exclusive; the sum of counts may exceed the number of respondents who reported any HRSN screening (n=57).

^b^Of 57 respondents who were screening for at least one HRSN.

^c^*P*<.05 for association with screening in bivariate analysis.

### Barriers to Screening

Among the 99 digital readiness survey respondents who were asked about perceived barriers to screening (Multimedia Appendix 1, Table S3), the most frequently cited challenges were lack of resources to address digital access (n=45, 45%), lack of resources to implement screening (n=42, 42%), and lack of time (n=41, 41%).

## Discussion

### Principal Findings

In this study on digital readiness screening, only 44% of respondents reported that their organizations screened for barriers to patient engagement with digital health tools. This is lower than the 58% who reported screening for other HRSNs in our sample and the 79% seen in a study of approximately 3000 US hospitals [25]. This gap may reflect a stronger infrastructure and policy focus on other HRSNs, particularly following the 2019 National Academies of Sciences, Engineering, and Medicine report on integrating social care into health care delivery [26]. For example, the Center for Medicare and Medicaid Services incentivizes HRSN screening but excludes digital readiness, reflecting the failure to recognize digital access as a social need and potentially limiting reimbursement and thereby exploration and awareness of strategies to address patients’ digital access and literacy challenges. Digital readiness was not associated with HRSN screening, suggesting that even organizations already engaged in social risk screening have not yet adopted digital readiness screening.

Given health care’s increasing reliance on patient portals, telehealth, and remote monitoring—and known disparities in adoption of digital health tools [27-30]—equity advocates worry that digitization intended to increase access may instead worsen disparities in access, engagement, and outcomes [31]. This screening gap represents a missed opportunity for health care organizations to address a barrier that directly impacts other HRSNs that are increasingly managed through digital platforms, such as access to food, housing, or transportation resources [32]. Moreover, without routine digital readiness screening, health systems and policymakers lack the data needed to quantify the scope of digital access barriers or track progress toward digital equity over time.

Organizations serving uninsured patients—populations most vulnerable to digital exclusion—were significantly less likely to screen for digital readiness. This highlights a critical paradox: organizations caring for patients most likely to face digital readiness barriers may also lack the resources or awareness needed to screen for them, as reflected by the frequently reported barriers to screening in our study (lack of resources to address needs, limited infrastructure for screening, and inadequate staff training). Current federal digital readiness policies do not target safety-net systems or address these operational challenges, reflecting a broader pattern of structural underinvestment in organizations that disproportionately serve patients at the highest risk for digital exclusion (ie, patients with LEP or lower income, or those in underconnected areas) [30,33-36]. Resource constraints (lower reimbursement rates, understaffing) limit capacity to train staff, implement screening, and build referral networks [37,38]. Addressing these disparities will require targeted policies that provide sustainable funding for digital access programs, reimbursement for screening and navigation, and investment in workforce training tailored to safety-net settings. Developing standardized digital readiness screening tools for integration into electronic health records, like those for other HRSNs, may reduce implementation barriers. Embedding digital screening into existing reimbursable activities and incorporating equity-focused metrics into value-based payment models could further promote adoption [39]. Additionally, health care systems could leverage existing reimbursement codes and build partnerships with community-based digital readiness organizations that provide skills training, device access, and connectivity support. Community health workers may also represent a mechanism to support digital readiness screening, although sustainable funding is lacking [40].

Awareness of digital equity policies was significantly associated with screening. However, overall awareness was low, with fewer than a quarter of respondents familiar with each of the 6 policies. Even among organizations accepting MA, only a minority were aware of the MA digital literacy screening requirement. These findings suggest that policy changes without dissemination, financial support, and institutional prioritization may be insufficient to drive adoption. There are similar gaps in policy dissemination to individual patients; in 2023, nearly 50% of eligible households were not aware of the ACP [41]. Ultimately, improved policy visibility, organizational engagement, and stable federal investment are necessary to promote digital readiness. However, ongoing policy uncertainties, including failure to ensure sustained availability of affordable internet service [22] and the cancellation of the Digital Equity Act [42], present threats to progress toward digital equity [43].

### Limitations

The use of convenience sampling may have limited generalizability, as organizations with a greater interest in digital access or HRSN screening may have been more likely to respond (eg, academic health systems and FQHCs). The causality of identified associations also cannot be determined from our observational data. While our survey specified that respondents should be able to comment on digital screening practices, we did not vet the individual qualifications to answer all survey questions. Therefore, as with any self-reported measures, there may have been unreliable responses. Despite these limitations, our study is strengthened by being one of the first assessments evaluating how health care systems are screening for digital readiness, with representation from a breadth of clinical care settings.

### Conclusion

This national survey found that digital readiness screening is not routinely conducted in US health care settings, especially among organizations serving populations most at risk for digital exclusion. Without intervention, patients already facing barriers to care risk being left further behind, exacerbating health disparities. As HRSN screening demonstrates, creating standardized and integrated digital readiness screening is feasible, but awareness and prioritization are lacking. Policy solutions that support safety-net systems, such as broadband subsidies, increasing device access, and improving digital navigation, may drive broader screening and intervention. Increasing awareness of existing digital equity initiatives is also needed to sustain progress and advocate for policy development. By identifying low policy awareness, associations between policy familiarity and screening, and implementation barriers, our findings offer actionable insights for those seeking to advance digital readiness screening.

## Appendix

Multimedia Appendix 1 Additional details on methods, Digital Inclusion Screening Survey Instrument, and Tables S1-S3.

## Abbreviations

ACP: Affordable Connectivity Program
FCC: Federal Communications Commission
FQHC: federally qualified health center
HRSN: health-related social need
LEP: limited English proficiency
MA: Medicare Advantage
OR: odds ratio
UCSF: University of California, San Francisco

## Data Availability

The datasets generated or analyzed during this study are available in the openICPSR repository [44].
