# Supplementary material for: Screening by Health Care Systems for Barriers to Patient Engagement With Digital Health Care: Cross-Sectional Survey Study
Source: JMIR Form Res. 2026 Feb 25;10:e85205. doi: 10.2196/85205 (PMC12980063; doi:10.2196/85205)
Supplement: Multimedia Appendix 1 [file formative_v10i1e85205_app1.docx]

**Supplementary Appendices**

Appendix A: Additional Methods Details
Appendix B: Digital Inclusion Screening Survey Instrument
Appendix C: Appendix Table 1. Sensitivity Analyses (Recoded Outcome): Characteristics of Screening among Overall Sample (N=144)

Appendix D: Appendix Table 2. Sensitivity Analyses (Recoded Outcome): Characteristics of Health-Related Social Needs Screening and Digital Inclusion Policy (N=99)

Appendix E: Appendix Table 3. Barriers to Screening for Digital Readiness (N= 99)

**Appendix A**: **Additional Methodological Details**

**Methods (Recruitment, Data Procedures)**

Recruitment:

To gather our sample, we first contacted primary care and ambulatory focused practice-based research networks listed on the Agency for Healthcare Research and Quality (AHRQ) Practice Based Research Network (PBRN) registry to ask for assistance in distributing our survey. We prioritized networks in the registry that were either comprised of >50 clinics, had a national presence or served multiple geographic areas (more than one U.S state), or had research interests in primary care. To increase our recruitment rate, we emailed members of various health-related professional networks (e.g., medical informatics, internal medicine, social interventions research, health equity). One of our team members also shared the survey link through their professional social media account (e.g., LinkedIn). In total, we recruited participants from eight distinct online sources.

Data Quality Review, Data Cleaning, and Data Merging:

Before conducting analyses, we implemented a step-by-step data cleaning protocol (described in detail below this section) to verify the quality of responses. This was initiated after noticing suspected “bot” activity from one of our survey links. We removed responses deemed fraudulent through a set of criteria our team believed indicated “bot” activity. Furthermore, we also reviewed responses that may have originated from the same organization or level of organization. When we encountered respondents in our survey from the same organizations, we retained the entry whose respondent was responding from the broadest level of the organization (e.g., entire health system vs. a single clinic/several clinics). On the other hand, we included all entries whose respondents were responding from seemingly mutually exclusive levels of the same organization (i.e., a single clinic vs. several clinics/inpatient care). We applied modified criteria for removing duplicate organizations in the other study team’s digital navigation survey since their respondents did not report the level of organization they were responding from. When there were respondents from the same organization, we kept the response from the individual whose title was best aligned with understanding the organization’s screening process (e.g., kept medical director, disregarded physician response). If the same organization was represented in both surveys, we retained the response from our digital readiness screening survey because it had the full list of questions. In total, we removed 7 duplicate entries from the digital readiness screening survey, 2 duplicate entries from the digital navigation survey, and 1 duplicate entry between the two surveys. Lastly, we removed 18 entries from the digital readiness screening survey that left all questions in our variable list blank.

We then merged the responses from the two surveys for the overlapping questions. However, there were differences between the two surveys in how we asked respondents to report their organization’s patient population. Our digital readiness screening survey asked respondents to report from this percentage breakdown (<1%, 1-<10%, 10-<25%, 25-<50%, ≥50%, Unsure/Don’t know) the approximate percentage of patients identified as racial or ethnic minorities or had limited English proficiency whereas the digital navigation survey asked respondents to report from a slightly different percentage breakdown (0-25%, 26-50%, 51-75%, 76-100%) the approximate percentage of patients identified in each race (African American/Black, Asian, Latinx, Native American, White, Other) or were non-English speakers. To align both of our coding schemes for the racial/ethnic breakdown responses, we first created a new variable and recoded the digital navigation survey’s racial and ethnic responses based on patients who identified as White and inversed those codes to elicit those who identify as racial or ethnic minorities (0-25% or 26-50% White recoded as ≥50% racial/ethnic minorities, 76-100% recoded as 0-< 25%, 51-75% recoded as 25-<50%). We then collapsed our responses <1%, 1-<10%, and 10-<25% to 0-<25%, and left 25-<50%, ≥50%, and unsure unchanged. For limited English proficiency, we created a new variable and recoded the digital navigation survey’s non-English speaker responses 0-25% as 0-< 25%, 26-50% as 25-<50%, and 51-75% or 76-100% as 50% or more, in addition to collapsing our responses <1%, 1- <10%, and 10- <25% to 0- <25%, and left 25- <50%, ≥50%, and unsure unchanged. It is important to note that through merging the demographic responses, the final percentage breakdowns (0- <25%, 25- <50%, ≥50%, Unsure/Don’t know) are close approximations as some of the cutoff points between the two breakdowns were not exact (e.g., 26-50% vs 25- <50%).

Survey Data Quality Review Protocol:

**Objective**

To remove test entries, non-consented responses, and invalid or bot-generated submissions, ensuring a valid, analyzable dataset.

**Raw Data Overview**

All survey responses, including entries from a known bot-infiltrated survey link, were consolidated into a single dataset for processing. The following multi-phase cleaning protocol was applied.

**Phase 1: Removing Test Entries and Non-Consented Responses**

Responses were excluded if they met any of the following criteria:

1. Survey link was intended as a preview (if Distribution Channel = preview)
2. Pilot responses submitted before survey was published (based on timestamps)
3. If respondent did not consent to participate (if Consent = No or = Blank for the required question)

**Phase 2: Removing Invalid or Bot-Generated Responses**

Two study team members (JJS, VEK) independently screened the dataset, reviewing each entry against the following criteria. Then, the reviewers met to resolve discrepancies and reached a consensus. A third study team member adjudicated unresolved cases (AY, RLE).

Responses were excluded if any one of these criteria were met:

1. Duplicate IP Addresses: Consecutive entries with identical IP addresses submitted within one hour. For cases with duplicate IPs but plausible response patterns, reviewers cross-checked free-text fields (e.g., organization name, job title, screening tool used) to determine validity.
2. Invalid Organization Name: Entries listing organizations unlikely to participate (e.g., “WHO,” “NIH”) or not representing a health system.
3. Rapid Sequential Responses: Consecutive entries (from any IP address) initiated within 10 seconds of each other and completed in similar durations (e.g., three responses started within 10 seconds, and each took around 800 seconds to complete, such as 800, 803, and 807 seconds).
4. Internal Inconsistency: Contradictory responses across variables (e.g., listing a private practice clinic but selecting “VA - Veteran Affairs” for clinic type). Each variable was cross-checked for consistency.
5. Bot-Like Free Text Patterns: Overly generalized, lengthy, and unusually well-punctuated free-text responses suggestive of automated generation.

**Appendix B: Digital Readiness Screening Survey Instrument**

# **Landscape Survey of Digital Health Screening among Health Systems/Organizations**

START OF BLOCK: DIGITAL HEALTH SCREENING

We are conducting research to understand how health organizations address the challenge of digitally connecting with patients. Please answer each question to the best of your ability.

1. Which of the following forms of virtual health services are offered by your health care organization? Select all that apply.

- Patient portal
- Video visits
- Phone visits
- Remote patient monitoring (e.g., telemonitoring of vital signs or other biometric information such as blood glucose)
- Secure bidirectional text messaging (e.g., not appointment text reminders)
- Other, please specify:
- None (Skip to Health System Characteristics Block)

1. Does anywhere in your organization screen patients for barriers to digital access or inclusion? This may include, but is not limited to screening for access to devices, internet, and/or skills necessary to participate in virtual health services such as patient portals or remote visits. [Also asked in digital navigation survey]

- Yes
- No (Skip to Q12)
- Not currently, but planning to (Skip to Q12)
- Don’t know (Skip to Q12)

1. In your organization’s digital inclusion screening, which of the following do you screen for? Select all that apply. [Also asked in digital navigation survey]

- Access to a digital device (e.g., smartphone, laptop, tablet)
- Internet access
- Technology skills/digital literacy
- Support from someone to assist with technology issues
- English language proficiency
- Educational level
- Cognitive ability
- Patient preference or interest in video visits, phone visits, or digital communication
- A private location conducive to a telehealth visit
- Need for accessibility services (e.g., hearing, vision, etc)
- Other, please specify:

1. Please indicate how and when you screen for barriers to digital inclusion. Select all the following options that apply to your organization’s clinical processes and workflow.

|  | Annually | As part of outreach unconnected to a clinic visit | As part of workflow connected to a clinic visit | | During new patient enrollment | | Not currently a practice |
| --- | --- | --- | --- | --- | --- | --- | --- |
| Patient completes online screening tool on their own at home or in the clinic (e.g., like through a patient portal) |  |  | |  | |  |  |
| Patient completes paper screening tool on their own at home or in the clinic |  |  | |  | |  |  |
| Clinical team member administers screening tool face-to-face |  |  | |  | |  |  |
| Clinical team member administers screening tool through telephone |  |  | |  | |  |  |
| Clinical team member screens patient based on information in electronic health record (e.g., age, language, non-use of portal) |  |  | |  | |  |  |
| Clinical team member informally screens patient without a screening tool (e.g., via conversation during visit) |  |  | |  | |  |  |

1. How does your organization record the digital inclusion screening results in your electronic health record (EHR)? Select all that apply.

- Entered in a structured field in EHR
- Documented in free text in EHR
- Tracked using another system outside EHR
- Other, please specify:
- Don’t know (Skip to Q8)
- Not formally tracked (Skip to Q8)

1. Does your organization periodically review the results of your digital inclusion screening?

- Yes
- No (Skip to Q8)
- Don’t know (Skip to Q8)

1. When you review the results of your digital inclusion screening, how do you use the results? (open-ended)
2. What is the source of your digital inclusion screening questions? Select all that apply. [Also asked in digital navigation survey]

- Standardized screening tool developed outside your health system (display Q9 if selected)
- Adaptation of a previously standardized screening tool (display Q9 if selected)
- Internally developed tool
- Our health system does not use a structured screening tool (skip to Q12)
- Don’t know
- Other, please specify:

1. What validated screening tool or adaptation of the validated tool do you use? (open-ended) [Also asked in digital navigation survey]
2. Have you collected feedback about your screening tool?

- Yes (Display Q11 if selected)
- No
- Don’t know

1. What type of feedback did you collect about the tool? How did you collect that feedback and from whom? Please list specific examples. (open-ended)
2. What barriers exist to screening for digital access/inclusion in your health organization? Select all that apply.

- Lack of time
- Lack of staff/clinician confidence or interest in screening
- Lack of resources to screen (e.g., EHR with integrated screening questions)
- Lack of resources to address identified barriers to digital access
- Belief that digital access is not a problem among our patients
- Staff not trained for digital access or inclusion screening
- Inadequate evidence of patient interest in virtual health services
- Other, please specify:
- Don’t know
- No barriers exist

END OF BLOCK: DIGITAL HEALTH SCREENING

START OF BLOCK: ADDRESSING CHALLENGES TO DIGITAL HEALTH ACCESS

1. How does your health organization assist patients who want to get a device to access virtual health services? Select all that apply.

- Directly give device to patients
- Directly loan device to patients
- Partner with community-based organizations to give or loan device to patients
- Provide patients with information about federal benefit programs (e.g., Affordable Connectivity Program) that subsidize purchasing of device
- Help patients apply for federal benefit programs that subsidize device purchase
- Partner with community-based organizations to help patients apply for federal benefit programs that subsidize device purchase
- Other, please specify:
- Don’t know
- We do not have workflows to address patient’s device needs

1. How does your health organization assist patients who want to get internet service to access virtual health services? Select all that apply.

- Directly offer device with prepaid data plans
- Directly loan device with prepaid data plans
- Partner with community-based organizations to loan device with a mobile data plan to patients
- Provide patients with information about federal benefit programs (e.g., Affordable Connectivity Program) that subsidize household internet plans
- Help patients apply for federal benefit programs that subsidize internet plans
- Partner with community-based organizations to help patients apply for federal benefit programs that subsidize internet plans
- Other, please specify:
- Don’t know
- We do not have workflows to address patient’s internet access needs

1. How does your health organization assist patients who want to increase their digital health literacy skills? Select all that apply.

- Directly provide ad-hoc in-person assistance with using technology to access healthcare
- Directly provide remote assistance (e.g., telephone) with using technology to access healthcare
- Offer basic training sessions on using technology to access healthcare
- Refer patients to tech support through their device’s company or the virtual health application’s team
- Partner with community-based organizations to provide digital health skill training
- Other, please specify:
- Don’t know
- We do not have workflows to address patient’s digital literacy training needs

1. In your health organization, who is in the telehealth support (i.e., digital navigator) role? Select all that apply. [Also asked in digital navigation survey]

- Medical assistant
- Front desk staff
- Nursing staff (e.g., RNs, LVN, CRNs)
- Prescribing clinician (e.g., physician, nurse practitioner, physician assistant, PharmD)
- Behavioral health staff (e.g., psychologists, MSWs, LCSWs)
- IT department
- Digital technology support staff
- Community health worker
- Nonprofit staff
- Government employee
- Librarian
- Other, please specify:

1. How familiar are you with the following policies?

|  | Have not heard of it | Have heard of it but not familiar | Familiar with the policy details |
| --- | --- | --- | --- |
| FCC's Affordable Connectivity Program |  |  |  |
| Health People 2030 health literacy objectives focused on health communication and information technology |  |  |  |
| Joint Commission Requirements to Reduce Health Care Disparities |  |  |  |
| Medicare Advantage digital literacy screening requirements |  |  |  |
| Digital Equity Act |  |  |  |
| Medicare Physician Fee Schedule Payment for Community Health Integration Services by Community Health Workers |  |  |  |

END BLOCK: ADDRESSING CHALLENGES TO DIGITAL HEALTH ACCESS

START OF BLOCK: HEALTH SYSTEM CHARACTERISTICS

1. What is the title of your role/position in your health organization? (open-ended) [Also asked in digital navigation survey]
2. What is the name of your health organization? (open-ended)
3. From what level of the organization are you responding about? Select all that apply.

- A single clinic
- Several clinics
- Inpatient care
- Entire health system/organization
- Other, please specify:

1. Which state(s) and/or U.S territory does your health organization serve? Select all that apply.
2. Which type of locations does your health organization serves. Select all that apply. [Also asked in digital navigation survey]

- Urban: Counties in metropolitan areas of 250,000 population to 1 million or more; Counties in metro areas of fewer than 250,000 population
- Suburban: Urban population of 2,500 to 20,000 or more, adjacent or not adjacent to a metro area
- Rural: Completely rural or less than 2,500 urban population, adjacent to a metropolitan area

1. Which of the following best describes the health organization that you are part of? Select all that apply. [Also asked in digital navigation survey]

- Academic
- Not-for-profit
- For-profit
- Non-federal governmental (e.g., state or county funded institution)
- Federally Qualified Health Center (FQHC)
- Veteran affairs (VA)
- Other, please specify:

1. What types of payors does your health organization accept? Select all that apply. [Also asked in digital navigation survey]

- Medicaid (e.g., MediCal in California)
- Traditional Medicare
- Medicare Advantage plan
- Commercial insurance plan
- City or county safety-net insurance (e.g., Healthy San Francisco)
- Uninsured / self-pay
- Don’t know
- Other, please specify:

1. Does your health organization currently participate in any value-based program (e.g., pay for performance, accountable care organizations, or Medicare shared savings program)?

- Yes
- No
- Don’t know

1. Please estimate the percentage of ambulatory care patients served by your health organization that fit these descriptions? These groups are the “Covered Populations” in the Digital Equity Act.

|  | <1% | ≥1 to  <10% | ≥10 to  <25% | ≥25 to  <50% | ≥50% | Unsure/ Don’t know |
| --- | --- | --- | --- | --- | --- | --- |
| Patients with limited English proficiency |  |  |  |  |  |  |
| Older adults (60yo+) |  |  |  |  |  |  |
| Residents in rural areas |  |  |  |  |  |  |
| Racial or ethnic minorities |  |  |  |  |  |  |
| Patients with physical or mental disabilities |  |  |  |  |  |  |
| Incarcerated individuals |  |  |  |  |  |  |
| Veterans |  |  |  |  |  |  |
| Individuals living in households with income below 150% of poverty level |  |  |  |  |  |  |

1. Does your organization routinely screen patients for social needs/social determinants of health (e.g., housing, food, transportation)?
   - Yes (Display Q28 if selected)
   - No
   - Not currently, but planning to
   - Don’t know
2. Please indicate which social needs/social determinants of health are routinely assessed. Select all that apply.

- Housing (instability, quality, financing)
- Food insecurity or hunger
- Utility needs
- Interpersonal violence
- Transportation
- Employment and income
- Education
- Social isolation (lack of family and social support)
- Other, please specify:

END OF BLOCK: HEALTH SYSTEM CHARACTERISTICS

END OF SURVEY (REDIRECT TO CONTACT INFO SURVEY)

**Appendix C: Appendix Table S1. Sensitivity Analyses (Recoded Outcome): Characteristics of Screening among Overall Sample (n=144)**

|  | **Respondents, No (%)** | |
| --- | --- | --- |
| **Characteristic** | **Number of respondents+**  n / 144 (% of total) | **Proportion that currently or plan to screen for digital readiness** |
| **Overall** |  | 81/144 (56%) |
| **Type of Health System** | | |
| Not for profit | 45/144 (31%) | 27/45 (60%) |
| Academic | 42/144 (29%) | 23/42 (55%) |
| Federally qualified health center (FQHC) | 40/144 (28%) | 18/40 (45%) |
| Non-federal governmental (e.g., state or county funded institution) | 19/144 (13%) | 12/19 (63%) |
| For profit | 18/144 (13%) | 11/18 (61%) |
| Veteran affairs (VA) | 6/144 (4%) | 5/6 (83%) |
| Other | 5/144 (4%) | 2/5 (40%) |
| **Location Types** | | |
| Urban | 96/144 (67%) | 52/96 (54%) |
| Suburban | 52/144 (36%) | 28/52 (54%) |
| Rural | 33/144 (23%) | 18/33 (55%) |
| **Payors Accepted** | | |
| Medicaid | 104/144 (72%) | 53/104 (51%) |
| Traditional Medicare | 101/144 (70%) | 50/101 (50%) |
| Commercial insurance plan* | 90/144 (63%) | 42/90 (47%) |
| Uninsured/ self-pay* | 88/144 (61%) | 41/88 (47%) |
| Medicare advantage plan | 87/144 (60%) | 46/87 (53%) |
| City or county safety-net insurance | 59/144 (41%) | 28/59 (48%) |
| Other | 10/144 (7%) | 5/10 (50%) |
| Don’t know | 3/144 (2%) | 1/3 (33%) |
| **Percent of Patients with Limited English Language Proficiency** | | |
| 0- < 25% | 58/144 (40%) | 31/58 (53%) |
| 25 - <50% | 29/144 (20%) | 17/29 (59%) |
| 50%+ | 24/144 (17%) | 11/24 (46%) |
| Unsure/Don’t know | 8/144 (6%) | 4/8 (50%) |
| **Percent of Patients that Identify as Racial/Ethnic Minority Populations** | | |
| 0- < 25% | 40/144 (28%) | 22/40 (55%) |
| 25 - <50% | 21/144 (15%) | 11/21 (52%) |
| 50%+ | 50/144 (35%) | 28/50 (56%) |
| Unsure/Don’t know | 8/144 (6%) | 2/8 (25%) |

^+^Percentages are calculated using the full sample of 144 respondents. Missing responses ranged from 19 to 25 across variables: health system type (19), location type (21), accepted payors (23), and patient demographics (25). Categories within health system type, location type, and payors accepted were not mutually exclusive, so sum of counts may exceed the total sample size.

*p < 0.05 for association with screening in bivariate analysis

**Appendix D: Appendix Table S2. Sensitivity Analyses (Recoded Outcome): Characteristics of Health-Related Social Needs Screening and Familiarity with Digital Readiness Policy (n=99)**

|  | **Respondents, No (%)** | |
| --- | --- | --- |
| **Characteristic** | **Number of respondents+**  n / 99 (% of total) | **Proportion that currently or plan to screen for digital readiness** |
| **Overall** |  | 58/99 (59%) |
| **Currently Screen for other Health-Related Social Needs (HRSN)** | | |
| Yes | 57/99 (58%) | 37/57 (65%) |
| No | 17/99 (17%) | 7/17 (41%) |
| **Screen for other Health-Related Social Needs (HRSN) Items ^a^** | | |
| Food insecurity or hunger | 51/57 (89%) | 32/51 (63%) |
| Housing (instability, quality, financing) | 48/57 (84%) | 31/48 (65%) |
| Interpersonal violence | 46/57 (81%) | 28/46 (61%) |
| Transportation | 39/57 (68%) | 24/39 (62%) |
| Employment and income | 35/57 (61%) | 21/35 (60%) |
| Social isolation (lack of family and social support) | 31/57 (54%) | 21/31 (68%) |
| Education | 29/57 (51%) | 17/29 (59%) |
| Utility needs* | 29/57 (51%) | 23/29 (79%) |
| Other | 3/57 (5%) | 3/3 (100%) |
| **Level of Familiarity with any of the six digital readiness policies** | | |
| Familiar with any policy details* | 47/99 (47%) | 35/47 (74%) |
| Not familiar with any policy details | 33/99 (33%) | 9/33 (27%) |
| **Level of Familiarity with the FCC’s Affordable Connectivity Program (ACP)** | | |
| Familiar with the policy details* | 24/99 (24%) | 21/24 (88%) |
| Not familiar with the policy details | 56/99 (57%) | 23/56 (41%) |
| **Level of Familiarity with the Joint Commission Requirements to Reduce Health Care Disparities** | | |
| Familiar with the policy details* | 20/99 (20%) | 16/20 (80%) |
| Not familiar with the policy details | 60/99 (61%) | 28/60 (47%) |
| **Level of Familiarity with the Medicare Physician Fee Schedule Payment for Community Health Integration Services by Community Health Workers** | | |
| Familiar with the policy details | 20/99 (20%) | 14/20 (70%) |
| Not familiar with the policy details | 60/99 (61%) | 30/60 (50%) |
| **Level of Familiarity with the Healthy People 2030 health literacy objectives focused on health communication and information technology** | | |
| Familiar with the policy details* | 18/99 (18%) | 14/18 (78%) |
| Not familiar with the policy details | 62/99 (63%) | 30/62 (48%) |
| **Level of Familiarity with the Digital Equity Act** | | |
| Familiar with the policy details* | 17/99 (17%) | 14/17 (82%) |
| Not familiar with the policy details | 63/99 (64%) | 30/63 (48%) |
| **Level of Familiarity with the Medicare Advantage digital literacy screening requirements** | | |
| Familiar with the policy details* | 17/99 (17%) | 14/17 (82%) |
| Not familiar with the policy details | 63/99 (64%) | 30/63 (48%) |

^+^Percentages are calculated using all 99 respondents who received these questions. Missing data ranged from 19 to 25 across items: policy familiarity (19) and HRSN screening behaviors (25). HRSN screening categories were not mutually exclusive; sum of counts may exceed the number of respondents who reported any HRSN screening (n=57).

^a^ Out of 57 respondents who are screening for at least one HRSN

*p < 0.05 for association with screening in bivariate analysis

**Appendix E: Appendix Table S3. Barriers to Screening for Digital Readiness (n= 99)**

| **Barriers to Screening** | **Total respondents with the characteristic^+^, No (%)** |
| --- | --- |
| Lack of resources to address identified barriers to digital access | 45 (45%) |
| Lack of resources to screen (e.g., EHR with integrated screening questions) | 42 (42%) |
| Lack of time | 41 (41%) |
| Lack of staff/clinician confidence or interest in screening | 37 (37%) |
| Staff not trained for digital access or inclusion screening | 33 (33%) |
| Inadequate evidence of patient interest in virtual health services | 12 (12%) |
| Other | 11 (11%) |
| Belief that digital access is not a problem among our patients | 8 (8%) |
| Don’t know | 6 (6%) |
| No barriers exist | 3 (3%) |

^+^Percentages are calculated using all 99 respondents who received this question. 16 did not report barriers to screening. Barriers to screening were not mutually exclusive; sum of counts may exceed the sample size of 99.
